# Surgery

**Published:** 1905-03

**Authors:** J. Lacy Firth


### SURGERY.

**The treatment of wounds of the thoracic duct** has recently been discussed by Erwin von Graff.[1] The paper is based on one case of the author's and the records of twenty-six cases collected from literature. In each case the duct was injured during an operation on the left side of the neck for the removal of malignant or tubercular lymphatic glands or of a malignant goitre. The author's case, operated upon by Prof. von Eiselsberg, was a child of 13. During the removal of tubercular glands near the left clavicle a large lymph-vessel was injured, probably the thoracic duct, and a free discharge of milky-white lymph occurred. The lymphatic vessel was ligatured, and the after course of the case was in no way prejudicially influenced by the accident or the treatment adopted. This case and a few others which have been treated in the same way, with equally good results, show that accidental injury of the thoracic duct need cause no grave anxiety. But in the great majority of cases the duct has not been ligatured; instead the wound has been packed, or no special treatment has been adopted. In these cases the flow of chyle generally continued for a long time, and injured the general health of the patients more or less severely. In two cases, those of Cheever and Schopf, death soon followed the operations. In the former of these the fatal termination was certainly not due to the loss of lymph, and in the other perhaps the loss of lymph should be regarded only as an important contributory cause of death. In all the other cases, even where the rapid loss of strength and great wasting of the patients gave rise to the gravest anxiety, there was a sudden change for the better as soon as the discharge of lymph ceased.

The absence of agreement with reference to the treatment of wounds of the thoracic duct can be explained in several ways. In the first place the injury is a rare one. The author's twenty-seven collected cases were published during a period of twenty-nine years, and in most of the scattered articles on the subject no definite conclusions, based upon the results of the various methods of treatment, have been drawn. Again, the accident often occurred at the end of a long and difficult operation, and the treatment by packing was chosen to save time;

[1] *Wien. klin. Wchnschr.*, 1905, xviii. 10.

also, by many, the ligature of the thoracic duct has been considered dangerous.

The fear of some authors that ligature of the thoracic duct might endanger life and lead to rupture of the distal end of the duct with the effusion of chyle in the chest or abdomen has been shown to be groundless by numerous experiments on animals and by anatomical researches in man. For example, it is known that the duct often opens into the innominate vein by many branches. Thus, in a case of Brinton's (Philadelphia), quoted by the writer, the thoracic duct in the upper part of the thorax divided into two branches, which again united with one another, forming a closed ring. From this ring three branches passed—one to the subclavian vein, another to the internal jugular, and a third passed first upwards and then obliquely to the left across the neck. It is obvious that there would have been no complete obstruction to the circulation of chyle by ligaturing one of the branches mentioned. Wendel[1] has specially investigated the collateral vessels communicating with the thoracic duct. He found that the duct gave off several branches at its upper end in 50 per cent. of the cases examined, and in two cases, by injecting the duct, he demonstrated the existence of small anastomotic communications between it and the azygos vein. Probably these collaterals, capable of dilatation, exist in all cases, so that the ligature of an unbranched thoracic duct in the neck will not prevent the flow of chyle into the venous system.

In sixteen of the twenty-seven cases of wounded thoracic duct collected by von Graff the treatment was by tamponade. In only three, or 9 per cent., of these cases was the treatment immediately successful. In the others the chylorrhœa continued for a time, and usually with injurious effects upon the patients' general health, and with great prolongation of the time required for the healing of the wound. In one case, for example, even three months after the operation, chyle was still being discharged from a fistula in the scar. In two of the thirteen cases in which chyle continued to escape in spite of the tamponade of the wound, no special change in the general health of the patients was noticed, though in one of them the quantity of chyle lost was large. In all the other cases severe disturbance of nutrition was produced by the loss of chyle.

To illustrate the clinical course of cases treated by tamponade the following examples may be cited:—Swinn, on changing the dressing on the tenth day in a case of excision of tubercular glands, noticed a tense elastic swelling immediately above the clavicle. Two days later, on removing the sutures, a great quantity of milky fluid escaped, which was proved by microscopical examination to be chyle. In spite of tight packing the fluid continued to escape, sometimes to the quantity of two

[1] *Deutsche Ztschr. f. Chir.*, xiviii., heft 5 and 6 (quoted by von Graff litres in twenty-four hours. The patient quickly lost weight, and suffered from severe headache with a frequent, weak pulse and serious fainting fits. The opening of the thoracic duct was therefore sought for and occluded with a clamp forceps. The flow of chyle was thus stopped, and the patient quickly regained his strength. In Wendel's case it was only on the nineteenth day that the free flow of chyle was checked, though the wound had been repeatedly and firmly packed. By this time the patient had become extremely weak, but from the moment the discharge was stopped he began to recover quickly.

The course of the twelve cases of wounding of the thoracic duct treated by ligature, clamping, or suture was far better than that depicted above. These methods were used at the time of the injury in nine cases, and subsequently, when tamponade had proved insufficient, in three cases. Keen, Cushing and Porter have each sutured a rent in the duct successfully. In the remaining cases the flow of chyle was checked by ligation or clamping. In nine, or 75 per cent., of these cases the chylorrhœa was checked immediately.

In the cases treated exclusively and successfully by tamponade, the cessation of the discharge of chyle after a certain time is easily comprehensible. The pressure of the packing by obstructing the outflow of chyle leads to the dilatation of the collateral branches of the duct. Finally all, or nearly all, the chyle flows to the blood-vessels through these dilated branches. When this occurs the wounded duct heals by granulation, which it could not do when its edges were separated and bathed by the freely-escaping chyle.

Erwin von Graff draws the following conclusions from the above considerations:—

1. Chylorrhœa causes severe but usually transient disturbances of nutrition. These can, in conjunction with other injurious influences, such as the loss of blood and prolonged anæsthesia, lead to a fatal result.

2. The only procedure which yields immediate success in treatment is ligature or suture of the injured duct, and this should be performed either immediately or, if that is impossible, secondarily.

3. The ligature of the duct produces no injurious effects on the system.

4. Tamponade is the next best treatment, but should only be used when ligature or suture is impossible.

E. Deanesly[1] records a case in which he divided the thoracic duct in removing tubercular glands, and implanted its proximal end in the internal jugular vein. The vein had been previously ligatured and divided near the innominate, and again ligatured higher up in the anterior triangle. The thoracic duct was

[1] *Lancet*, 1903, ii. 1783.

grafted into the portion of the vein lying between the high and the low ligatures, after it had been proved that blood circulated in this part of the vein, probably from the establishment of collateral circulation through thyroid veins. The patient had no untoward symptoms of any kind. Charles R. Keyser[1] records a case in which, after an operation for the removal of carcinomatous glands from the supra-clavicular region on the left side, there was a copious discharge, amounting to several pints, of creamy fluid from the wound. The discharge continued for a fortnight, and then gradually ceased.

**The treatment of incontinence of urine.** In 1903 Fernand Cathelin, of the Hôpital Necker, Paris, introduced a method of treating incontinence of urine by epidural injections. The injections are made into the lowest part of the sacral canal through the inverted V-shaped notch at the inferior and posterior border of that bone. For making the injections the patient is placed conveniently in the Sims' position with the knees drawn well up towards the chest. A clear description of the technique is given by Ferd. C. Valentine[2] and again by F. Dejardin.[3] The last perceptible sacral spinous process is made out with the left index and middle fingers which are then slightly separated and pushed downward until each rests upon an osseous tubercle. The skin is stretched with these fingers, and the needle passed in between them. The needle is first held at an angle of about 40° to the surface, but when its point is felt to have penetrated the posterior-inferior sacral obturating membrane the shaft of the needle is depressed about 20° and cautiously pushed up into the sacral canal. The fluid used by Valentine was decinormal salt solution, and that used by Dejardin a solution of bicarbonate of soda 1 % and sodium chloride 7 %. The injections are made slowly, 5 c.c. per minute, and from 5 to 20 c.c. of fluid used. Valentine reports the results in eight patients. All were inmates of the Manhattan Hospital West, and were of unsound mind and affected with enuresis or incontinence or both. In all gratifying results were obtained. Case 2 may be mentioned as an example. Before epidural injections the patient urinated into bed ten times nightly and into her garments six times daily. After the tenth injection she no longer wetted either her bed or clothing. The operation is not more painful than hypodermic injection. Generally there are no ill effects observable, but sometimes the patients feel a sense of pressure in the spine, others become dizzy, others have moderate pain along one or other sciatic region, and a few have had transient painless paraplegia. The longest time any of these effects lasted was about five minutes.

[1] *Lancet*, 1904, ii. 1215. [2] *Med. Rec.*, 1903, lxiv. 486.
[3] *Arch. prov. de Chir.*, 1904, xiii. 513

Valentine considers the following deductions warranted:—(1) That epidural injections offer the most promising results in abnormalities of urination due to faulty vesical innervation; (2) that incontinence of urine, enuresis, excessive frequency of micturition (unless due to other pathological conditions), can at least be ameliorated by the injections; (3) that they are in no wise dangerous; (4) that they are but slightly painful; (5) that the technique is simple; (6) that the immediate effects are very rarely even disagreeable.

Dejardin[1] fully reviews this subject, and reports six cases of ordinary incontinence in children he has treated with success by the method; also he has tried the method for the treatment of rectal prolapse with success. The action of these injections seems to be inhibitory, the shock produced at the level of the nerve roots being transmitted gradually to the centres themselves; in fact, a fracture of the sacrum may be associated with the same effects as epidural injections.

J. LACY FIRTH.

### LARYNGOLOGY AND RHINOLOGY.

[1] *Loc. cit.*

